# Dynamical transitions during the collapse of inertial holes

**DOI:** 10.1038/s41598-019-50956-w

**Published:** 2019-10-10

**Authors:** Jiakai Lu, Carlos M Corvalan

**Affiliations:** 1 Department of Food Science, University of Massachusetts, Amherst, MA 01003 USA; 2 Transport Phenomena Laboratory, Department of Food Science, Purdue University, West Lafayette, IN 47907 USA

**Keywords:** Chemical engineering, Fluid dynamics

## Abstract

At the center of a collapsing hole lies a singularity, a point of infinite curvature where the governing equations break down. It is a topic of fundamental physical interest to clarify the dynamics of fluids approaching such singularities. Here, we use scaling arguments supported by high-fidelity simulations to analyze the dynamics of an axisymmetric hole undergoing capillary collapse in a fluid sheet of small viscosity. We characterize the transitions between the different dynamical regimes —from the initial inviscid dynamics that dominate the collapse at early times to the final Stokes dynamics that dominate near the singularity— and demonstrate that the crossover hole radii for these transitions are related to the fluid viscosity by power-law relationships. The findings have practical implications for the integrity of perforated fluid films, such as bubble films and biological membranes, as well as fundamental implications for the physics of fluids converging to a singularity.

## Introduction

The dynamics of a small hole in a thin sheet of fluid play a central role in a range of natural and physical systems, such as the evolution of perforated bubble films and biological membranes^1,2^, the stability of bubbles and foams in the food industry^3^, the generation of ocean mist from bursting bubbles^4^, and the production of sprays from fragmented liquid sheets^5^. Recently, the controlled contraction of microscopic holes in fluidized plastic and silicon sheets has enabled a host of analytical applications, including pore-based biosensors for rapid characterization of DNA and proteins^6–9^. Moreover, the dynamics of a contracting hole in a liquid sheet is analogous to that observed during the final stages of bubble pinch-off, when the neck of the bubble collapses, and therefore relevant to the control of bubble size and growth-time in a variety of applications, including microfluidics^10^, and the production of microbubbles in food processing and medicine^11^.

The initial size of the hole largely determines the subsequent evolution. If the size is large in relation to the sheet thickness, the hole expands driven by capillary forces, leading to the fragmentation of the liquid sheet^5,12^. For low-viscosity liquids, the dynamics of expansion are characterized by a constant terminal velocity known as the Taylor-Culick velocity^13,14^. More recently, Savva and Bush^15^ extended the inertial studies to include the effect of the liquid viscosity, showing that after an initial stage of exponential expansion, which is influenced by both the sheet geometry and the liquid viscosity, the hole reach the terminal Taylor–Culick velocity predicted for the inertial case. On the other hand, if the hole size is small in relation to the sheet thickness, the hole contracts, and the integrity of the sheet is preserved^13^. Besides their practical importance for the integrity of liquid films, the dynamics of collapsing holes is of fundamental fluid mechanics interest because it represents an archetypal example of free-surface flow with a finite-time singularity. In this case a singularity of the surface curvature that occurs at the center of the collapsing hole.

Other free-surface flows with singularities, such as drop and filament breakup, exhibit temporal transitions between inertial and viscous regimes as the system approaches the singularity^16–19^. Such dynamical transitions have also been observed via careful visualization experiments during the collapsing stage of bubble-pinchoff ^20–23^, which have features in common with collapsing fluid holes and share the same dynamics^24–27^. But the extent of the different dynamical regimes during hole collapse, and the crossover hole size for the transitions have not yet been fully characterized. Here, we use scaling arguments supported by high-fidelity simulations to gain detailed insight into the dynamical transitions of a low-viscosity fluid hole undergoing capillary collapse. If the fluid viscosity is sufficiently low, the hole exhibits two dynamical regimes, an inviscid regime that dominates at early times and a Stokes regime that dominates near the singularity, with an intermediate region between them. By following the hole collapse for more than four decades in size, results from the simulations demonstrate that the extent of the inviscid, intermediate, and Stokes regimes can be understood from a force-balance analysis. Moreover, scaling arguments enable the development of power laws to estimate the crossover hole size for the transitions between the different regimes.

## Free-Surface Model and Method

**Figure 1:**
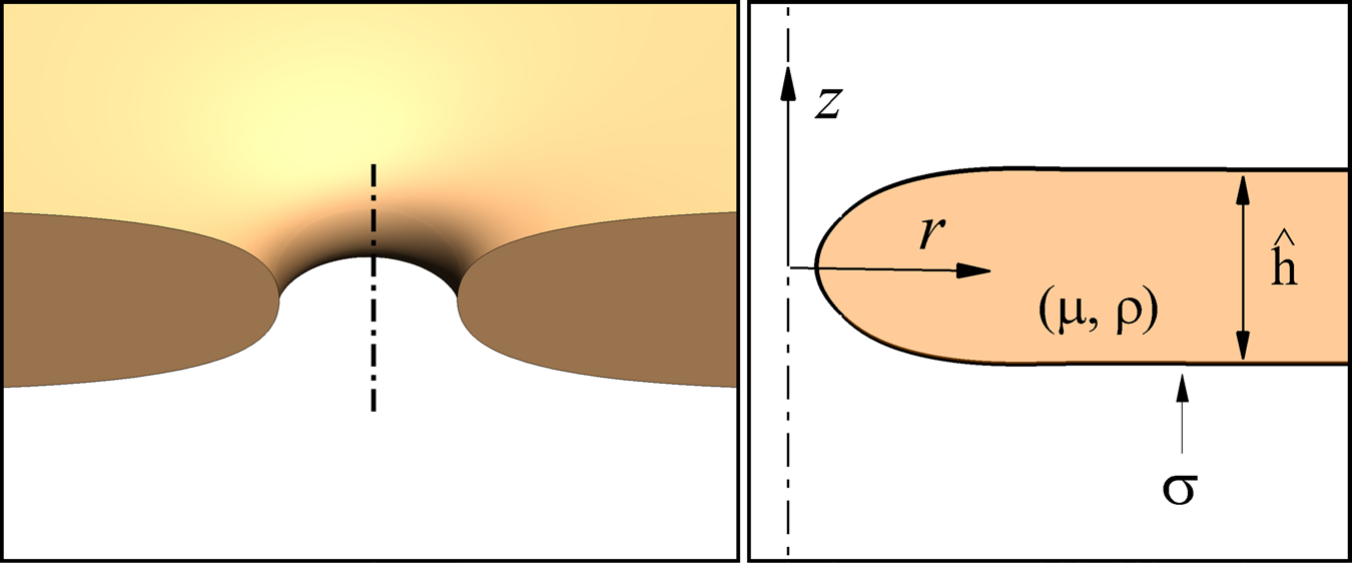
Definition sketch for studying the dynamics of a small axisymmetric hole in a fluid of density *ρ*, viscosity *μ*, and surface tension *σ*. The initial radius of the hole is ${\hat{r}}_{0}$, and the thickness of the fluid sheet is $\hat{h}$.

The free-surface dynamics of collapsing holes are studied here by following the spatial and temporal evolution of a circular toroidal hole of small initial radius ${\hat{r}}_{0}$ in a liquid sheet of density *ρ*, viscosity *μ*, and surface tension *σ*, as sketched in Fig. 1. The flow dynamics is characterized by solving the full axisymmetric continuity and Navier-Stokes equations for the velocity **v** and pressure *p*,

$$\nabla \cdot {\bf{v}}=0,$$

$$\frac{\partial {\bf{v}}}{\partial t}+{\bf{v}}\cdot \nabla {\bf{v}}=O{h}^{2}\,\nabla \cdot {\bf{T}},$$

where **T** = −*p***I** + *τ* is the Cauchy stress tensor, and *τ* = [∇**v** + (∇**v**)^*T*^] the viscous stress tensor. Gravity is considered negligible for the small pores, and the surrounding air is considered a dynamically inactive fluid. The model is cast into dimensionless form using the initial sheet thickness *ĥ* as characteristic length scale, and the capillary velocity (*σ*/*μ*) as velocity scale. Using these scales, relevant dimensional parameters for the circular pore are the initial pore size *r*_0_ = ${\hat{r}}_{0}$/*ĥ* and the Ohnesorge number *Oh* = *μ*/(*ρσĥ*)^1/2^. Along the free phase interface both the traction boundary condition

$${\bf{n}}\cdot {\bf{T}}=2H{\bf{n}}$$

and the kinematic boundary condition

$${\bf{n}}\cdot ({\bf{v}}-{{\bf{v}}}_{s})=0$$

are imposed, where **n** is the unit vector normal to the interface, **v**_*s*_ the velocity of the points on the interface, and *H* = ∇_*s*_ · **n** the local mean curvature of the interface, with ∇_*s*_ = (**I** − **nn**) · ∇ the surface gradient operator^28^. In addition, symmetry boundary conditions are imposed on the center line, and on the plane *z* = 0 (Fig. 1).

The free-surface Navier-Stokes system and associated boundary conditions are solved using the finite-element method for spatial discretization along with the arbitrary Lagrangian-Eulerian method of spines to trace the deforming interface as detailed in^29^, and adaptive time integration as described in^30^. The resulting discretized system of equations is solved simultaneously for velocity, pressure and location of the interface using a Newton’s method with full analytical Jacobian^29^. The Newton iterations are continued until the *L*_2_ norm of the residuals for all the independent variables falls below ≈10^−6^, and the time steps are adaptively chosen using first-order continuation as implemented in^31^ to improve computational efficiency. Mesh independence studies were carried out at various resolutions, and meshes with degrees of freedom between approximately 15000 and 20000 degree of freedom were selected depending on the *Oh*. We have previously benchmarked and successfully applied this numerical scheme to simulate similar free-surface flows, including drop coalescence^32^, breakup of Newtonian and non-Newtonian liquid filaments^33^, collapse of viscous and inertial pores^34,35^, and contraction of surfactant-laden pores and filaments^36,37^.

## Results and Discussion

**Figure 2:**
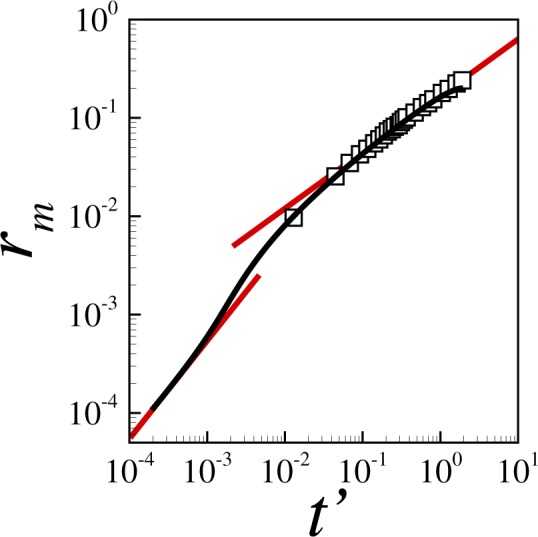
Evolution of minimum hole radius *r*_*m*_ with time to collapse *t*′ during the pinchoff of a circular hole in a slightly viscous liquid with *Oh* = 0.066. Simulations (black line) and experiments by Thoroddsen *et al*.^20^ (symbols) show an initial regime in which *r*_*m*_ = (*Oht*′)^0.57^ (top red line), and a final regime in which *r*_*m*_ = 1/2 *t*′ (bottom red line).

We begin our discussion by summarizing the various dynamics of contraction in Fig. 2. The figure illustrates the contraction of the small cavity formed during the pinch-off of a bubble in a slightly viscous liquid with Ohnesorge number *Oh* = *μ*/(*ρσĥ*)^1/2^ = 0.066. This system has been previously characterized experimentally using high-speed visualization^20^, and can thus also been used as a benchmark. Results show that the simulations (black line) agree well with the experiments (symbols). Moreover, in the early stages of contraction, the experiments confirm the existence of an inertial regime (top red line) in which the data approximately follow the rate of collapse expected for an inviscid liquid^20,34,38^. Similarly, in the vicinity of pinch off, when the minimum hole radius *r*_*m*_ → 0, the dynamics enters a final viscous regime (bottom red line) in which the data follow the theoretical rate of collapse corresponding to a Stokes liquid^21,24,35^. The results are also consistent with the findings in^23^. Using both theory and visualization experiments, Bolanos *et al*.^23^ shows that for fluids with intermediate viscosities the scaling *r*_*m*_ ∼ *t*′^*α*^ cannot describe the full contraction event with a single scaling exponent because, as shown in Fig. 2, the value of the exponent changes with time from the value of the initial inertial regime (*α* ≈ 0.57) to the value of the late Stokes regime (*α* = 1).

**Figure 3:**
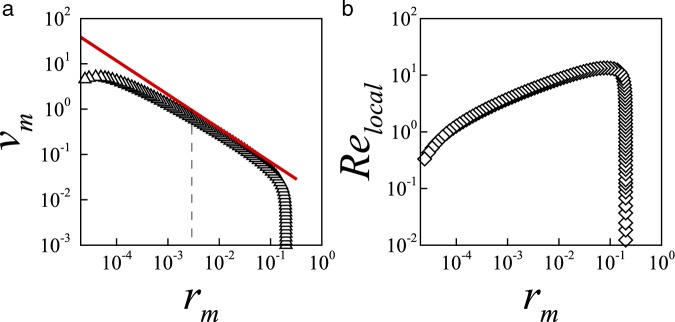
Results showing the temporary nature of the inviscid regime. (**a**) Evolution of hole velocity *v*_*m*_ with minimum hole radius *r*_*m*_ in a low-viscosity liquid sheet with *Oh* = 0.018. Despite the small *Oh*, the velocity gradually moves away from the inviscid scaling *v*_*m*_ = *αOhr*_*m*_^1−1/*α*^ (Eq. 1, red line) as *r*_*m*_ → 0. The dashed line identify the hole radius at which *Ca*^*I*^ = 1 (Eq. 5). (**b**) Evolution of the local Reynolds number *Re*_*local*_ as a function of *r*_*m*_. The initial hole radius is *r*_0_ = 0.2.

Having shown that the solution of the full Navier-Stokes system confirms limiting theoretical scalings and agrees well with experiments, we now discuss the rate of contraction of low-viscosity holes to quantify the transitions between the dynamical regimes. Figure 3a illustrates the evolution of the rate of contraction *v*_*m*_ with minimum hole radius *r*_*m*_ for a low-viscosity hole of *Oh* = 0.018. As expected for this small Ohnesorge number, the hole velocity follows the scaling

1 $${v}_{m}=\alpha Oh\,{r}_{m}^{1-1/\alpha },$$

where *α* ≈ 0.57, indicating that the contraction initiated by surface tension is now dominated by inertia^20,34,38^. Indeed, for the contraction of an axisymmetric cavity in a fluid of negligible viscosity, Eggers *et al*.^38^ demonstrated, using asymptotic theory, that a logarithmic correction to a leading order value 1/2 yields a slowly varying exponent *α*, for which they determined an approximate effective value *α* = 0.559 from a fit to their numerical data. This value is close to and explains typical values in the range *α* = 0.57 ± 0.03 reported from detailed experiments and simulations^20,34,39,40^. Figure 3a shows that the agreement of the hole velocity (symbols) with the inertial scaling (red line) is excellent but, by following the contraction of the hole radius for about four orders of magnitude, it also makes clear that the inertial scaling does not continue all the way to collapse. Despite the small *Oh*, the inertial scaling only describes the early dynamics, and the hole velocity gradually moves away from the inviscid regime as the hole contracts.

The short life of the inviscid regime can be verified directly by defining a local Reynolds number

2 $${R}{{e}}_{local}\equiv \frac{\rho {\hat{v}}_{m}{\hat{r}}_{m}}{\mu }=\frac{1}{O{h}^{2}}{v}_{m}{r}_{m},$$

which characterizes the relative importance of inertial and viscous forces in the vicinity of the meniscus tip. According to Eq. 1, in the inviscid regime the local Reynolds number should scale with hole radius *r*_*m*_ as

3 $${\mathrm{Re}}^{I}=\frac{\alpha }{Oh}\,{r}_{m}^{2-1/\alpha }.$$

Because *α* ≈ 0.57 > 1/2, the *Re*^*I*^ → 0 as the hole radius *r*_*m*_ → 0, confirming that the dynamics of an inertial hole cannot remain in the inviscid regime as the hole collapses. Therefore, as the inertial hole contracts, viscous forces should eventually become sufficiently strong to impact the evolution of the pore, and a potential switch of the dynamics to a viscous regime when $R{e}^{I} \sim 1$ has to be considered when approaching the singularity.

To gain more information about the hole dynamics, we plot in Fig. 3b the calculated *Re*_*local*_ as a function of *r*_*m*_. The main findings depicted in Fig. 3b are twofold. First, the figure makes clear that, after a short transient, the $R{e}_{local}\gg 1$ at early times, further confirming the initial inertial dynamics observed in Fig. 3a. Second, by the time the local Reynolds has fallen to *Re*_*local*_ ≈ 1, the growing hole velocity *v*_*m*_ reaches a maximum and start to decrease, indicating that the dynamics has switched to a regime in which both the inertial forces and the opposing viscous forces are relevant. Below the radius corresponding to the maximum velocity (*r*_*m*_ ≈ 5 × 10^−5^), the calculated *Re*_*local*_ decreases at increasingly growing rate but a clear switch to the viscous dominated regime cannot be observed within the limits of the figure.

**Figure 4:**
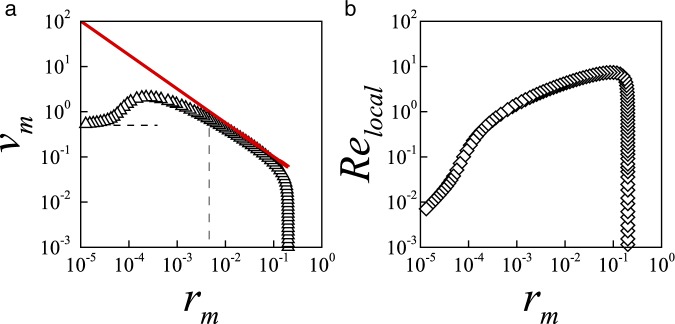
Results showing the inception of a late Stokes regime in a inertial pore. (**a**) Evolution of hole velocity *v*_*m*_ with minimum pore radius *r*_*m*_ in a low-viscosity liquid sheet with *Oh* = 0.032. The velocity initially follows the inviscid scaling *v*_*m*_ = *αOhr*_*m*_^1−1/*α*^ (Eq. 1, red line) and eventually approaches the Stokes velocity *v*_*m*_ ≈ 1/2 (horizontal dashed line) as *r*_*m*_ → 0. (**b**) The local Reynolds number confirms the inception of a late Stokes regime $R{e}_{local}\ll 1$ as *r*_*m*_ → 0. The initial radius is *r*_0_ = 0.2.

Together, Fig. 3a,b show that the collapsing hole departs from the inertial dynamics well before the local Reynolds has fallen to *Re*_*local*_ ≈ 1 due to the presence of an intermediate region. In addition, in the inertial dynamics viscous forces are small relative to both inertial and capillary forces. Consequently, in the inertial dynamics the local Reynolds number *Re*_*local*_ > 1 and the local Capillary number *Ca*_*local*_ < 1, where

4 $$C{a}_{local}\equiv \mu {\hat{v}}_{m}/\sigma ={v}_{m}$$

represents the relative importance of viscous and capillary forces in the vicinity of the leading edge. The observed transition from inertial to intermediate dynamics is consistent with the theoretical threshold *Ca*^*I*^ ∼ 1 for the local Capillary number in the inviscid regime, *Ca*^*I*^ = *α* *Ohr*_*m*_^1−1*/α*^ (see Eq. 1), which estimates the end of the inertial regime as

5 $${r}_{m}\sim {(\alpha Oh)}^{\alpha /(1-\alpha )}$$

for fluids with $Oh\ll 1$. For illustration, the crossover radii estimated by Eq. 5 are shown as dashed lines in Figs 3 and 4.

Having demonstrated the temporary nature of the inertial regime, we now consider in Fig. 4 the dynamics of an inertial hole which shows evidence of a transition to the viscous regime despite the fact that $Oh\ll 1$. The figure illustrates the computed variation of the hole velocity (Fig. 4a) and local Reynolds number (Fig. 4b) in a low-viscosity liquid sheet of *Oh* = 0.032. The figure shows that, after a short transient, the hole contracts in the inviscid regime where both ${v}_{m}\sim {r}_{m}^{1-\mathrm{1/}\alpha }$ and $R{e}_{local}\gg 1$, although the extent of the inviscid regime is shorter than for the sheet with *Oh* = 0.018. Indeed, the extent of the inviscid region shortens rapidly as the viscosity becomes increasingly important (Eq. 5), and our simulations show that when *Oh* increases above *Oh* ≈ 0.1 the inertial scaling is no longer observed. Importantly, the figure shows that near the end of the computations, when *r*_*m*_ ≈ 10^−5^, the calculated $R{e}_{local}\ll 1$ indicating the onset of the viscous regime anticipated by the theoretical scaling of Eq. 3.

Further confirming the incipient transition to the viscous dominated regime, Fig. 4a shows that when *r*_*m*_ ≈ 10^−5^ the rate of contraction slows down, and the velocity gradually stabilizes around *v*_*m*_ ≈ 1/2. This is significant because in a series of careful experiments conducted in highly-viscous liquid sheets, small holes were observed to contract at constant velocity^8,41^. Specifically, in the limit $Oh\gg 1$, when inertial forces are negligible, small cavities contract in a Stokes regime in which the force balance between viscous forces and capillary forces leads to a constant velocity of contraction *v*_*m*_ = 1/2^21,24,35^. The implication is that, despite $Oh\ll 1$, the inertial hole in the low-viscosity liquid sheet of Fig. 4 eventually adopts the constant velocity of contraction characteristic of highly-viscous liquid sheets.

**Figure 5:**
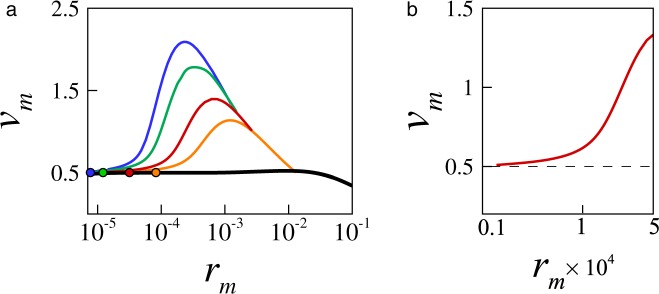
The dynamics of inertial holes (*Oh* < 0.1) transition to the Stokes regime as the holes collapse. (**a**) Velocity of contraction *v*_*m*_ as a function of *r*_*m*_ over a range of small Ohnesorge numbers *Oh* = 0.032, 0.035, 0.045 and 0.057 (from top to bottom). The black line corresponds to a highly-viscous hole with *Oh* = 1.4, and the solid circles identify the hole radius at which *Re*^*I*^ = 1 (Eq. 6). As *Oh* decreases, the hole velocity merges on the Stokes solution at smaller *r*_*m*_. (**b**) A finer level of detail suggests that the inertial pores approach the Stokes solution asymptotically. The initial hole radius is *r*_0_ = 0.2.

In Fig. 5a we summarize results for a series of inertial holes (*Oh* < 0.1) that exhibit a transition to the viscous regime within our range of data. For each Ohnesorge number, the transition to the viscous regime can be identified by following the velocity of contraction *v*_*m*_ as *r*_*m*_ → 0 until the velocity merges on the Stokes solution (black line). The figure clearly shows that as *Oh* decreases the dynamics transition to the viscous regime at a progressively smaller radius. Moreover, the observed transitions are consistent with Eq. 3, which for the theoretical threshold $R{e}^{I}\sim 1$ predicts a cross-over radius

6 $${r}_{m}\sim {(Oh/\alpha )}^{\alpha \mathrm{/(2}\alpha -\mathrm{1)}}$$

for holes with $Oh\ll 1$. For comparison, the cross-over radii predicted by Eq. 6 are shown as full circles in Fig. 5a. As would be expected, the predicted cross-over radii agree with the observed transitions for $Oh\ll 1$, but depart from the observed transitions for Ohnesorge numbers larger than *Oh* ≈ 0.1, where the pores are no longer inertial and Eq. 6 is no longer relevant. However, note that at a higher level of detail, the exact cross-over radius cannot be readily identified because the hole velocity approaches the Stokes solution asymptotically, as illustrated in the enlarged view in Fig. 5b.

**Figure 6:**
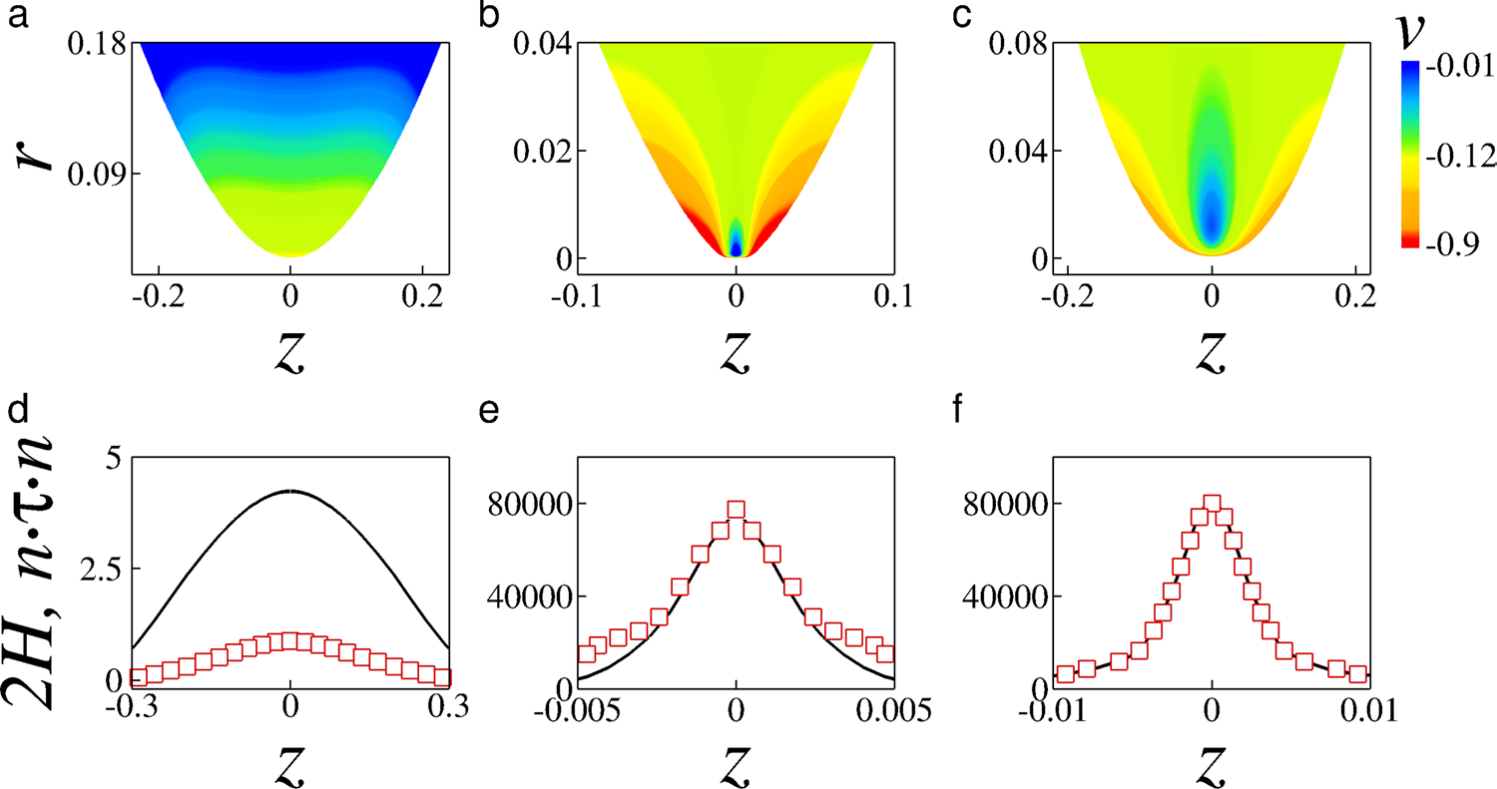
Flow field, capillary stress 2*H* (black solid line), and viscous normal stress **n** · *τ* · **n** (red symbols) near the meniscus front for a low-viscosity hole with *Oh* = 0.035 in (**a**,**d**) the initial inertial regime (*r*_*m*_ ≈ 3 × 10^−2^) and (**b**,**e**) the late viscous regime (*r*_*m*_ ≈ 1.2 × 10^−5^). Flow field (**c**) and stresses (**f**) are also shown for a viscous hole with *Oh* = 1.4 for comparison. The initial hole radius is *r*_0_ = 0.2.

The transition to the Stokes regime below the crossover radius is also reflected in the overall flow field. This is exemplified in Fig. 6a,b for the case of a low-viscosity hole with *Oh* = 0.035. The figures show the cross-sectional velocity field near the meniscus front both in the initial inertial regime (Fig. 6a) and in the late viscous regime (Fig. 6b). Because viscous forces are comparatively weak, in the initial inertial regime the velocity profiles are flat and essentially follow the gradient of capillary pressure as shown in Fig. 6a. Driven by the capillary forces 2*H* shown in Fig. 6d (black line) and opposed by the still weaker viscous stresses **n** · *τ* · **n** (symbols), the low viscosity fluid is continuously accelerated toward the meniscus tip. Later, as the hole size decreases and the hole dynamics enters the viscous regime, the flow pattern becomes drastically different as shown in Fig. 6b. Here, the velocity profiles are no longer flat because the opposing viscous forces reduce the flow velocity near the meniscus tip where viscous stresses are locally stronger. We also note in Fig. 6b that for this small radius the meniscus front becomes more slender in the vicinity of the tip due to the comparatively slow growth of the axial curvature, as observed in experiments by^20^. The significance of the viscous stresses is further illustrated in Fig. 6e, which shows that the magnitude of the viscous normal stress near the meniscus tip (symbols) balances almost exactly the capillary force at the interface (black line) resulting in the hole collapsing with uniform radial velocity. For comparison, we also show in Fig. 6c,f the case of a viscous hole with substantially larger *Oh* = 1.4. Clearly, the velocity field for the viscous fluid in Fig. 6c and the velocity field for the low-viscosity fluid in the viscous regime in Fig. 6b are qualitatively similar, although the length scale in Fig. 6c is significantly larger because the characteristic viscous length scale increases rapidly as *Oh* increases. For this larger *Oh* the inertial forces are small, and the dynamics is dominated by the balance of viscous stress against surface tension (Fig. 6f), which is qualitatively similar to what is observed in Fig. 6e for the low *Oh* case in the late viscous regime.

## Conclusion

We have used force-balance arguments supported by simulations to characterize the crossover between dynamical regimes during the capillary collapse of low-viscosity fluid holes. Our results estimate the transition from the inertial regime that dominates the dynamics at early times to an intermediate regime, and show that the estimated crossover hole size follows a direct power-law relationship with the viscosity. This power-law relationship helps explain why the inviscid regime shortens rapidly as the viscosity increases, and is not longer observed for fluid holes with moderate viscosities.

Moreover, as a manifestation of the singularity at the center of the hole, the dynamics eventually transitions from the intermediate region to a final Stokes regime where viscous forces dominate. Our results characterize the transition to the final Stokes regime for fluids with different viscosities, and show that the crossover hole size can be estimated by a power-law of the viscosity. This direct power-law relationship with the viscosity helps explain why the Stokes dynamics occurs at length-scales that cannot be readily interrogated experimentally as the fluid viscosity decreases.

Although our simulations solve the full Navier-Stokes system of governing equations, the results are limited by several simplifying assumptions, particularly the assumption of constant material properties. We expect that future studies will generalize this work to account for local changes in viscosity induced by non-Newtonian effects, and local changes in surface tension induced by the presence of surfactants, which may play a critical role on the dynamical transitions.
